# Self-supervised denoising of grating-based phase-contrast computed tomography

**DOI:** 10.1038/s41598-024-83517-x

**Published:** 2024-12-31

**Authors:** Sami Wirtensohn, Clemens Schmid, Daniel Berthe, Dominik John, Lisa Heck, Kirsten Taphorn, Silja Flenner, Julia Herzen

**Affiliations:** 1 Research Group Biomedical Imaging Physics, Department of Physics, TUM School of Natural Sciences, Technical University of Munich, 85748 Garching, Germany; 2 Chair of Biomedical Physics, Department of Physics, TUM School of Natural Sciences, Technical University of Munich, 85748 Garching, Germany; 3 Munich Institute of Biomedical Engineering, Technical University of Munich, 85748 Garching, Germany; 4 Institute of Materials Physics, Helmholtz-Zentrum Hereon, 21502 Geesthacht, Germany; 5 Present Address: Paul Scherer Institute, Forschungsstrasse 111, 5232 Villigen, Switzerland

**Keywords:** Computed tomography, X-ray imaging, Noise reduction, Self-supervised learning, Phase contrast, Data processing, X-rays, Biomedical engineering

## Abstract

In the last decade, grating-based phase-contrast computed tomography (gbPC-CT) has received growing interest. It provides additional information about the refractive index decrement in the sample. This signal shows an increased soft-tissue contrast. However, the resolution dependence of the signal poses a challenge: its contrast enhancement is overcompensated by the low resolution in low-dose applications such as clinical computed tomography. As a result, the implementation of gbPC-CT is currently tied to a higher dose. To reduce the dose, we introduce the self-supervised deep learning network Noise2Inverse into the field of gbPC-CT. We evaluate the behavior of the Noise2Inverse parameters on the phase-contrast results. Afterward, we compare its results with other denoising methods, namely the Statistical Iterative Reconstruction, Block Matching 3D, and Patchwise Phase Retrieval. In the example of Noise2Inverse, we show that deep learning networks can deliver superior denoising results with respect to the investigated image quality metrics. Their application allows to increase the resolution while maintaining the dose. At higher resolutions, gbPC-CT can naturally deliver higher contrast than conventional absorption-based CT. Therefore, the application of machine learning-based denoisers shifts the dose-normalized image quality in favor of gbPC-CT, bringing it one step closer to medical application.

## Introduction

X-ray computed tomography (CT) has become an invaluable tool for medical diagnosis. In Germany alone twelve million examinations are carried out per year^1^. It allows fast 3D visualization of the internal structure of the human body. The contrast is based on the attenuation of the X-rays passing through an object. Like conventional X-ray radiography, X-ray CT provides good contrast for dense materials like the mineralized structures of bone. However, the efficacy of traditional CT imaging encounters limitations when it comes to soft tissue examination. Soft tissues with similar X-ray attenuation properties often yield results with diminished contrast, posing a challenge for diagnoses^2^. In instances where medical assessments hinge on the visualization of high soft tissue contrast, alternative imaging modalities, such as Magnetic Resonance Imaging (MRI), are favored for their enhanced capability to discriminate soft tissue details at the cost of longer acquisition times and lower resolution.

The soft tissue contrast can be improved by the use of phase-sensitive imaging techniques. These methods are sensitive to the refractive index decrement in the sample, as demonstrated at high brilliant X-ray sources^3,4^. Due to the high cost and spatial requirements of synchrotron facilities, they are not feasible for medical applications. Therefore, the grating-based phase-contrast imaging (gbPCI) approach was developed to bring phase-contrast imaging to low brilliant X-ray sources^5^. The method shows promising results in improving soft-tissue contrast^2^, and delivers information about the differential phase and the small angle scattering of X-rays in the sample in addition to the attenuation. First, studies using ex-vivo samples showed improved visualization of lesions and microcalcifications for 2D grating-based phase-contrast mammography^6–9^. To exploit the contrast advantages of phase-sensitive imaging and the 3D information of CT, research advanced in the direction of grating-based phase-contrast computed tomography (gbPC-CT).

However, the gbPC-CT suffers from a main drawback preventing its medical application. Due to the integration of the differential phase, the reconstructed volume suffers from low-frequency noise, leading to a resolution-dependent performance relative to attenuation-based CT (abCT)^10,11^. For many medical applications like full-body CTs, the contrast advantages of the phase information can therefore be overcompensated by the lower spatial resolution, leading to an equal or even worse Contrast-to-Noise Ratio (CNR) per dose^11,12^. Additionally, the higher noise power in the low-frequencies of the gbPC-CT reduces the perceived image quality even at equal CNRs since it is more likely to interfere with the sample structure than the high-frequency noise of abCT^12^.

An increase in spatial resolution is required to take full advantage of the enhanced soft tissue contrast of the phase information in medical applications. However, a higher spatial resolution increases the dose since a certain CNR is necessary for clinical application. Hence, a denoiser is needed, which can drastically increase the CNR of high-resolution low-dose data and which is also able to effectively handle low-frequency noise to compensate for its impact on the observer performance.

In recent years, denoising techniques based on deep learning networks have been developed, showing promising results in medical applications^13–15^. Most of these networks need to be trained on a set of data containing noise-free images, but such data is not yet available for gbPC-CT. Therefore, we introduce the self-supervised deep learning network Noise2Inverse (N2I) into the field of gbPC-CT, which can be trained on a single noisy tomogram alone^16,17^. We also investigate the effects of its hyperparameters on the image quality. The performance of N2I is compared to other denoisers, namely Statistical Iterative Reconstruction (SIR), Block Matching 3D (BM3D), and Patchwise Phase Retrival (PPR). This work also introduces a qualitative evaluation system based on several known Image Quality Assessment (IQA) methods to quantify the results. Furthermore, the potential impact of deep learning neural networks, like N2I, on the field of gbPC-CT is investigated by measuring changes in the noise power spectrum.

## Methods

### Data acquisition

For clinical application, the most common X-ray source is a polychromatic X-ray tube. Therefore, all experiments are conducted at a rotating anode.

#### Experimental setup

The *MicroMax HF007* rotating anode with a molybdenum target from the *Rigaku Corporation* served as the source. For use in CT, an accelerating voltage of $50\, \hbox{kV}$ and a current of $24\, \hbox{mA}$ were chosen. The highest visibility is achieved for a distance between $G_0$ and $G_1$ of $l = {1080}\,\hbox {mm}$ and between $G_1$ and $G_2$ of $d = {518}\,\hbox {mm}.$ For the source grating $G_0$ a period of $p_0 = {10}\,\hbox {mm}$ and a $h_0 = {35}\,\hbox {mm}$ gold filling is used. The phase grating $G_1$ is a $\pi$-shifting grating with a period of $p_1 = {6.48}\,\hbox {mm}$ and a $h_1 = {9.5}\,\hbox {mm}$ nickle filling. For the analyzer grating $G_2$ a gold filling height of $h_2 = {50}\,\hbox {mm}$ and a period of $p_2 = {4.8}\,\hbox {mm}$ is utilized. The setup reaches a measured visibility of 11.5%. The sample is placed $85\, \hbox{mm}$ away from the $G_1$ and the detector is $20\, \hbox{mm}$ behind the analyzer grating $G_2$. For all measurements, the *Santis 0808 prototype* gallium arsenide photon counting detector of the *Dectris AG* is used. It has two vertically stacked $500\, \hbox{mm}$ thick modules with a pixel size of $75\, \hbox{mm}.$ All measurements are 5 x 5 binned, resulting in an effective pixel size of $281.5\, \hbox{mm}$. The pixel size is chosen to closer match the resolution used in medical imaging, at around $300\, \hbox{mm}$^18,19^.

#### Sample

For the investigation of denoising behavior, a phantom based on a marbled piece of pork neck is used. The phantom has a size $2.5 \times 2.5 \times 3\, \hbox{cm}$ and is placed in a Falcon tube with a 70% ethanol concentration after fixation in a 4% formaldehyde solution for one week. A polymethylmethacrylate (PMMA) rod is added for possible energy calibration. The whole tube is placed into a $3\, \hbox{cm}$ thick water bath to avoid strong phase changes at the tube-air boundary, resulting in an increased image quality^20,21^.

#### Signal retrieval

**Figure 1:**
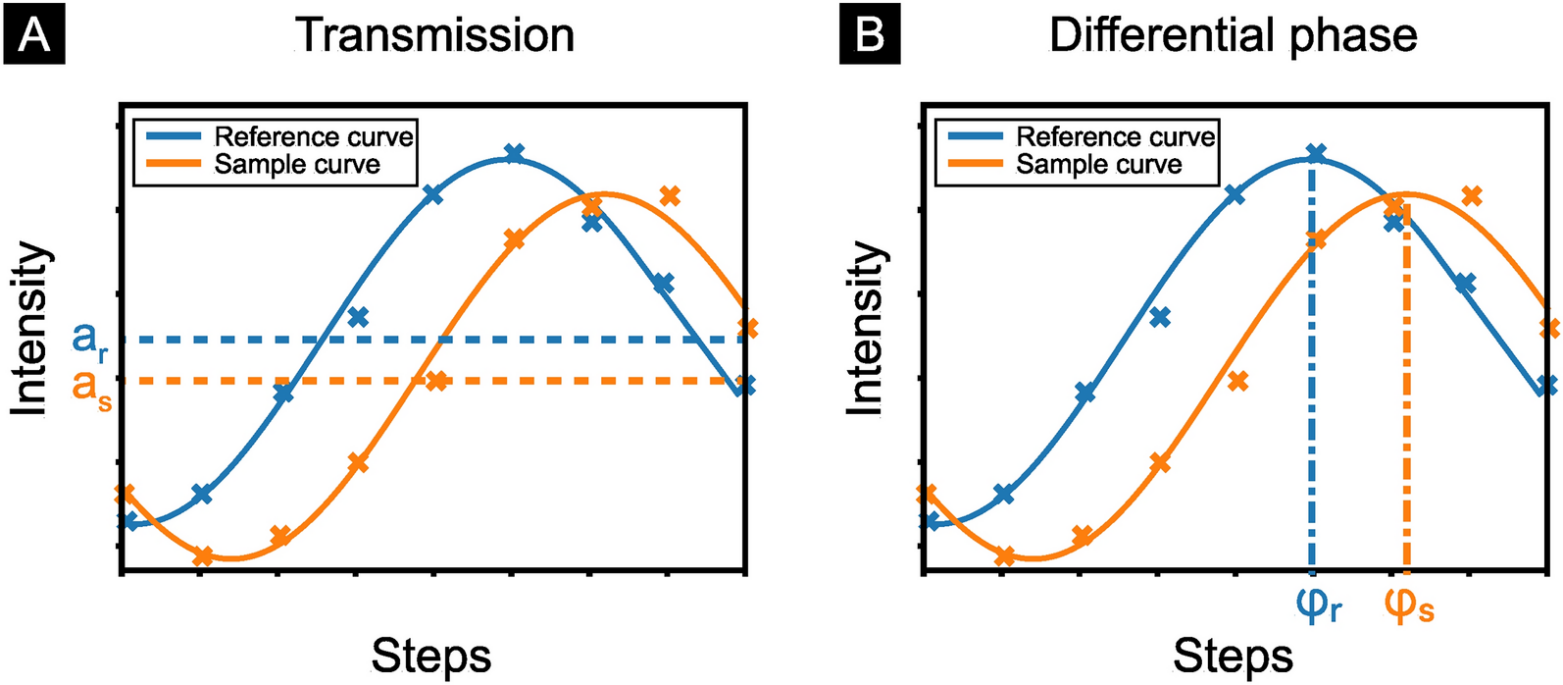
Signal retrieval process for the transmission (**A**) and differential phase (**B**) for a grating-based setup. The phase grating is stepped over one full grating period once with the sample in the beam (orange curve) and one without (blue curve). By plotting the intensity values of a single pixel over the stepping position, the stepping curve is created. The reduction of the mean intensity by the sample corresponds to the transmission signal (**A**), while the relative shift of the stepping curve gives information about the differential phase (**B**).

For each projection angle, the phase grating is laterally stepped over one full grating period. At each step, an image is taken. The pixel-wise intensity over the grating steps forms a sinusoidal stepping curve as seen in Fig. 1. The attenuation in the sample leads to a reduction of the mean intensity and shifts the stepping curve vertically (see Fig. 1 a). The transmission is therefore given by

1 $$\begin{aligned} T = \frac{a_{\text {s}}}{a_{\text {r}}}, \end{aligned}$$

with the mean sample intensity $a_{\text {s}}$ and the mean reference intensity ${a_\text {r}}$. The transmission contrast is identical to the one measured with a conventional X-ray set-up^22^.

The refractive index decrement in a sample leads to a lateral shift of this curve^22^. By fitting and comparing the sample curve to a reference curve measured without the sample in place, the differential phase contrast can be retrieved by

2 $$\begin{aligned} \varphi (x) = \varphi _{\text {s}} -\varphi _{\text {r}}, \end{aligned}$$

where $\varphi _s$ is the fitted phase coefficient of the sample and $\varphi _{\text {r}}$ of the reference stepping curve as shown in Fig. 1 b.

For all measurements, the stepping curve is created with five grating steps. However, if the total photon count for the stepping curve is fixed, the variance in the differential phase is independent of the number of grating steps^23^. This is valid as long as the sinusoidal curve is sufficiently sampled, which is the case for at least four equidistantly distributed grating steps. Hence, the experimental results are independent of the chosen number of grating steps besides the mentioned limitations.

If not other stated, the 3D volume of the attenuation coefficient is reconstructed based on a filtered back projection (FBP) using a Ram-Lak filter. To obtain the electron density and thus the phase contrast, the differential phase signal $\varphi (x)$ has to be integrated. This is performed during the 3D reconstruction by utilizing a Hilbert filter. Due to the integration process, the noise propagation changes, resulting in a higher noise power in the low-frequencies of the electron density compared to the attenuation coefficient. It is hereby not relevant if the integration takes place in the projection space or during reconstruction^24,25^. For both signals, the Python implementation of *X-AID* is used^26^.

#### Dose estimation

The mean glandular dose (MGD) is calculated based on the monoenergetic normalized glandular dose coefficients *DgN*(*E*, *t*, *g*) listed by Boone et al.^27^. The air kerma (kinetic energy released per unit mass) is measured with a Diados T60005 MAM clinical dosimeter (*PTW Freiburg GmbH*, Freiburg, Germany) at the sample position with installed gratings. The air kerma is defined by the initial kinetic energy $E_{kin}$ of all charged particles liberated by an incoming uncharged ionizing radiation per unit mass of air *m*

3 $$\begin{aligned} K = \frac{dE_{\text {kin}}}{dm}. \end{aligned}$$

The energy-dependent dose coefficient and the air kerma are multiplied with a conversion factor $\kappa = {0.114}\, \hbox {R/mGy}$ and summed over the energy bins of the source spectrum^28^

4 $$\begin{aligned} \text {MGD} = \sum _{E=E_{\text {min}}}^{E_{\text {max}}} \text {DnG}(E) \cdot K(E) \cdot \kappa . \end{aligned}$$

Based on the sample composition, a glandular composition of 50% is assumed. For the noisy measurements, a dose of $20\, \hbox{mGy}$  is chosen based on the upper limit for a typical whole-body CT examinations in Germany^29^. The experimental parameters of all measurements are listed in Table 1.

**Table 1:** Experimental scan parameter for all measurements.

Dose	Method	Projections	$t_{\text {exp}}$
20 mGy	FBP, SIR, BM3D	170	0.122 s
	N2I	340	0.061 s
1231 mGy	FBP	255	5.0 s

Each projection is retrieved from five grating steps and each grating step has an exposure time $t_{\text {exp}}$.

### Denoising methods

**Figure 2:**
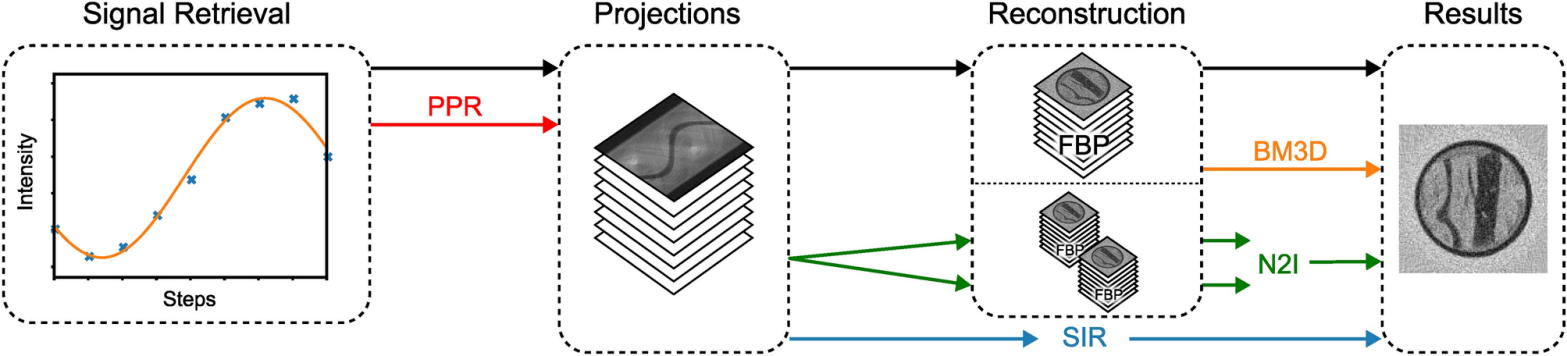
Flowchart of the general processing pipeline. Starting from the measured pixel-wise stepping curve intensities (left), the Patchwise Phase Retrieval (PPR) (red arrow) is applied during the phase retrieval on each projection. Statistical Iterative Reconstruction (SIR) (blue arrow) takes the signal-retrieved projections as input and returns the reconstructed volume. For Noise2Inverse (N2I) (green arrows), the projections are split into subsets, reconstructed individually, and used as input and target for training. N2I returns a full stack of denoised slices. Block Matching 3D (BM3D) (orange arrow) is applied on the reconstructed volume. For the PPR, N2I, and BM3D, the reconstruction is performed by a Filtered Back Projection (FBP) with a Ram-Lak filter.

In the following, all used denoising methods are briefly introduced. Figure 2 shows the general processing pipeline, including the interaction points of the used denoising methods.

#### Patchwise phase retrieval

PPR takes advantage of the signal-retrieving process of gbPCI to denoise the outputs. This is achieved by considering the information of the neighboring pixels. The phase retrieval algorithm fits a sine curve to the intensity values of all grating steps pixel-wise. The additional information of the neighboring pixels reduces the variance. It therefore improves the fitting accuracy resulting in a reduction of noise in the final image, if the sample is homogeneous in the neighboring region. But if the sample changes considerably in the neighborhood of a pixel, the PPR leads to blurring artifacts, which reduce the image quality. Hence, the included number of neighboring pixels must be chosen carefully and adjusted to the properties of the sample^30^.

#### Block matching 3D

BM3D is an image denoising algorithm that uses collaborative filters^31^. This is done by grouping similar 2D fragments of an image and stacking them into a 3D array. Since high similarity of small image fragments at different spatial locations is common in natural images, the transformation achieves a high sparse representation of the true signal and the noise can therefore be separated during shrinkage^31,32^. The sparsity increases with the number of grouped blocks. BM3D is proven to be an effective denoiser in the field of X-ray imaging^13,33,34^. We use a BM3D implementation based on the work of Y. Mäkinen et al.^35^.

#### Statistical iterative reconstruction

The SIR algorithm is a statistical reconstruction approach. It introduces a statistical model that describes the fluctuations in the measurement due to noise by taking advantage of known information about the volume and by using an iterative algorithm that approaches the correct image in multiple steps. Hence, it can also decrease the impact of sparse data and missing projection artifacts, and can handle complex geometries. In this paper, we use the python implementation of *X-AID*^26^.

#### Noise2Inverse

N2I is a self-supervised CNN-based denoising method, which does not need additional clean or noisy data for training^16^. It is therefore able to learn from one noisy measurement itself. N2I achieves that by dividing the noisy data $\textbf{x}$ into multiple subsets in which the noise is element-wise independent and zero-mean. It is analytically shown, that training a network on complementary noisy subsets $\textbf{x}_{J}$ and $\textbf{x}_{J^C}$ as input and target is as good as training the network on the ground truth $\textbf{z}_{J}$ up to a certain factor, which corresponds to the variance of the noise (see (7))^16,36^:

5 $$\begin{aligned} \mathbb {E}\Vert f(\textbf{x}_{J^C})_J - \textbf{x}_{J} \Vert ^2&= \mathbb {E}\Vert f(\textbf{x}_{J^C})_J - \textbf{z}_J + \textbf{z}_J - \textbf{x}_{J} \Vert ^2 \end{aligned}$$

6 $$\begin{aligned}&= \mathbb {E}\Vert f(\textbf{x}_{J^C})_J - \textbf{z}_J\Vert ^2 + \mathbb {E}\Vert \textbf{z}_J - \textbf{x}_J\Vert ^2 + 2\mathbb {E}_{\textbf{z}} \langle \mathbb {E}_{\textbf{x}|\textbf{z}}(f(\textbf{x}_{J^C})_J-\textbf{z}_J), \underbrace{\mathbb {E}_{\textbf{x}|\textbf{z}}(\textbf{z}_J-\textbf{x}_J)}_{= 0\text { if }\mathbb {E}[\textbf{x}|\textbf{z}]=\textbf{z}}\rangle \end{aligned}$$

7 $$\begin{aligned}&= \underbrace{\mathbb {E}\Vert f(\textbf{x}_{J^C})_J - \textbf{z}_J\Vert ^2}_\text {ground truth loss} + \underbrace{\mathbb {E}\Vert \textbf{z}_J - \textbf{x}_J\Vert ^2}_\text {variance of noise}. \end{aligned}$$

This makes N2I a possible solution for medical imaging applications, especially in fields where only limited data is available and therefore supervised learning approaches fail.

We applied N2I with the X:1 training strategy, in which all but one subsets are used as input, and the last subset is used as a target during training. As network architecture, the MS-D Net is employed^37^. All measurements N2I is trained and applied on are oversampled by a factor of two, meaning the measurement was taken with two times the number of angles recommended by the Nyquist-Shannon theorem. The recommended number of angles $N_{\text {ang}}$ based on the extend of the sample in pixels $N_{\text {pix}}$ are given by^38–40^:

8 $$\begin{aligned} N_{\text {ang}} = \frac{\pi N_{\text {pix}}}{2}. \end{aligned}$$

We also examine how the number of splits, the number of subsets into which the sinogram is divided, affects image quality.

**Figure 3:**
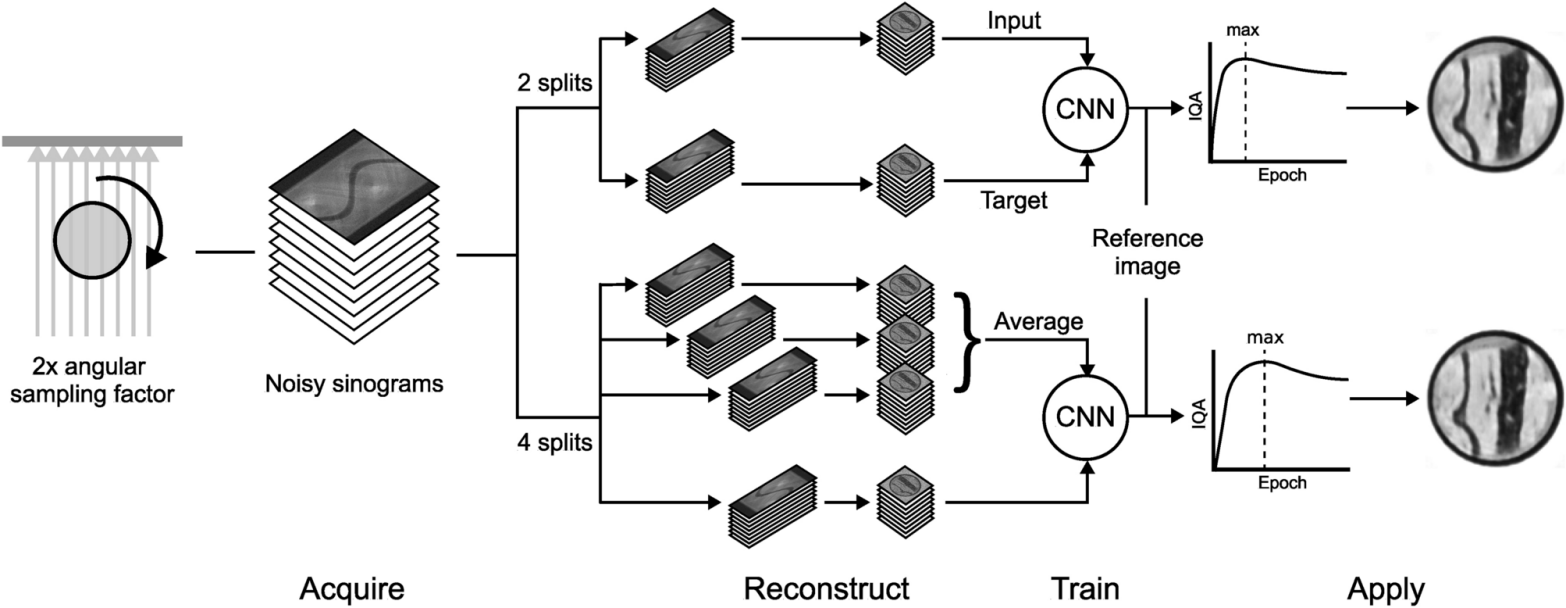
Flowchart of the processing pipeline for N2I. After the acquisition of the projections with a sampling factor of two (left), the noisy sinogram is split into multiple subsets. Each subset is then individually reconstructed and fed into N2I as input or target. For the used X:1 strategy the average of all but one subset is used as input and the last subset is used as target as shown in the case of four splits.

The training is performed on all available slices instead of splitting them into training and validation data. The reason is that the laboratory conditions allow us to take high-dose reference images, which are used to determine the optimal number of epochs by calculating and maximizing the IQA values, preventing the overfitting of noise. A new model is trained for each stack of slices per image modality. The model is afterward only applied to the training data itself; overfitting of present structures is therefore less of a concern. Figure 3 shows the processing pipeline for N2I once for two and once for four splits.

### Image quality assessment

To quantitatively compare the impact of different denoising algorithms, reliable image quality assessment metrics are required. In the following, the considered methods are explained.

#### Peak signal to noise ratio

A simple quality estimation can be achieved by looking at the mathematical difference of an estimator to its true value^41^. The normalized root mean squared error (NRMSE) compares two images by calculating the voxel-based error. It is based on the root-mean-square error (RMSE) which is given by

9 $$\begin{aligned} \text {RMSE} = \sqrt{\frac{1}{NM}\sum _{i=0}^{M}\sum _{j=0}^{N}\left( x_{ij} - y_{ij}\right) ^2}, \end{aligned}$$

where the image $x_{ij}$ and the reference image $y_{ij}$ both have the size *M* times *N*. In reality, the quality of an image is impacted through the presence of noise. Since the image quality perception of humans is not linear; we use the peak signal-to-noise ratio (PSNR). It measures the ratio between the maximum possible signal power and the noise power. It is defined as

10 $$\begin{aligned} \text {PSNR} = 20\log _{10}\left( \frac{\Vert {\textbf {y}}\Vert _{\infty }}{\text {RMSE}}\right) , \end{aligned}$$

where $\Vert \cdot \Vert _{\infty }$ denotes the supremum norm. However, the PSNR sometimes lacks to fully represent the human visual perception^42^. Therefore, we include a second image quality metric.

#### Structural similarity index

Since human vision is highly adapted to extract structural information, perceptual quality is closely related to it. The structural similarity (SSIM) index exploits this fact by comparing the structural degradation of an image to its reference to determine the perceptual quality change^43^. It is defined as:

11 $$\begin{aligned} \text {SSIM}(\textbf{x}, \textbf{y}) = \frac{\left( 2\sigma _x \sigma _y + C_1 \right) \left( 2\mu _x \mu _y + C_2\right) }{\left( \sigma _x^2 + \sigma _y^2 + C_1\right) \left( \mu _x^2 + \mu _y^2 + C_2\right) }, \end{aligned}$$

with the local means $\mu _x$ and $\mu _y$, the standard deviations $\sigma _x$ and $\sigma _y$, and the cross covariance $\sigma _{xy}$ for the test image $\textbf{x}$ and the reference image $\textbf{y}$. The variables $C_1$ and $C_2$ stabilize the division with weak denominators. It delivers values between 0 for a low and 1 for a high structural similarity^41^.

#### Edge preservation ratio

The edge preservation ratio (EPR) is an image sharpness assessment method based on the ratio of the extracted edges of a reference and a distorted image^44^. The detection of the edges can be performed through any edge detector. As suggested by^44^, this work uses the Canny filter *C* to determine the edge map based on local maxima of the gradient magnitude of a Gaussian-smoothed image^45^

12 $$\begin{aligned} R&= C(\textbf{y}, \textbf{p}), \end{aligned}$$

13 $$\begin{aligned} D&= C(\textbf{x}, \textbf{p}). \end{aligned}$$

*R* is the set of pixels representing edges in the reference image ${\textbf {y}}$, and *D* is the set of edge pixels in the distorted image $\textbf{x}$. Here $\textbf{p}$ describes the width of the Gaussian filter and the low and high hysteresis threshold. They are kept identical for the reference and distorted image. The EPR is subdivided into two sub-metrics, the EPR accuracy (EPRa) and the EPR robustness (EPRr). They are calculated by

14 $$\begin{aligned} \text {EPRa}&= \frac{|R\cap D|}{|R|}, \end{aligned}$$

15 $$\begin{aligned} \text {EPRr}&= \frac{|R\cap D|}{|D|}, \end{aligned}$$

where $|\cdot |$ denotes the number of edge pixels. The EPRa is a metric for edge preservation and is close to one for good edge conservation. The EPRr measures falsely introduced structures and is closer to one for less false structures^44^.

## Experimental results

### Noise2Inverse for phase-contrast imaging

In the initial N2I paper, Hendriksen et al.^16^ showed that two splits deliver good results in general, but the PSNR of conventional abCT can be improved by choosing four splits if the projection angles are not under-sampled. To find out whether this is also valid for the electron density of gbPC-CT, N2I is applied to a measurement with an angular sampling factor of two at $20\, \hbox{mGy}$, thus using two times as many projection angles as recommended by the Nyquist-Shannon theorem. The projections are then split once into two and once into four subsets, which are reconstructed independently. N2I is trained on each full set of subsets and for each signal type separately. The outputs are compared to a reference measurement performed at a high dose of $1231\, \hbox{mGy}$.

**Figure 4:**
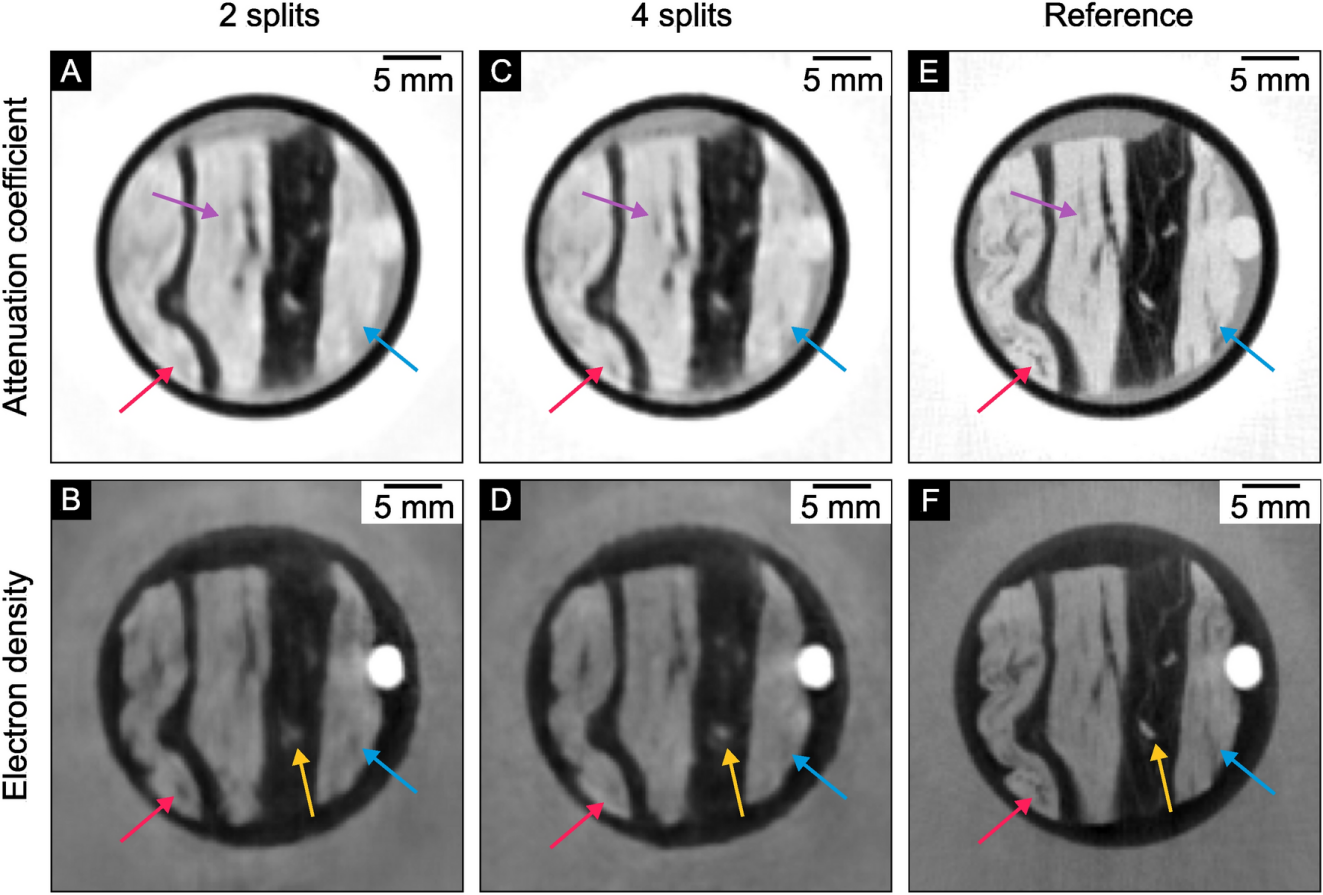
N2I result comparison of two and four splits at $20\,\hbox {mGy}$. The results of N2I with two (**A**, **B**) and four (**C**, **D**) splits at $20\,\hbox {mGy}$ and a high dose reference measurement at $1231\,\hbox {mGy}$ (**E**, **F**). For the electron density, the two splits (**B**) show more details in the muscle region marked with the red and blue arrow, while four splits (**D**) have a higher contrast for the small muscle structure in the lipid tissue marked with a yellow arrow. For the attenuation coefficient, the use of four splits (**C**) reveals more structures in the muscle tissue marked by the red, purple, and blue arrows. The windowing for the attenuation coefficient is: [-3.0, -0.5] x 10^-3^; for the electron density: [-1.5, 1.5] x 10^-2^.

The left column in Fig. 4 shows the results for the attenuation coefficient. N2I can retrieve small structures better using four splits. In the bottom left of the sample (red arrow), a small fiber in the muscle tissue is nearly indistinguishable in the case of two splits but can be seen in the result of four splits. This can also be observed in the bottom right region (blue arrow). The structure in the central muscle region (purple arrow) is also better recovered using four splits. For this reason, it is recommended to use four splits for the application to the attenuation coefficient. This result coincides with the findings of Hendriksen et al.^16^.

On the contrary, for the electron density, a closer examination of the right column in Fig. 4 reveals that the application with two splits retrieves more details in the muscle structure, which is visible in the left and right fibers (red and blue arrows). Therefore, separating the projections into two splits is preferred for the electron density.

### Denoiser quality comparison

In the following, different denoising techniques are compared. For that, four consecutive measurements were performed, all with five grating steps and three of the measurements with a dose of $20\,\hbox {mGy}$. The fourth one—the reference measurement—is again performed at a high dose of $1231\,\hbox {mGy}.$

For BM3D, a region of interest containing only the background is chosen manually and its standard deviation is calculated. This value is then used as input. BM3D is applied after reconstruction on the axial slices. Based on the previous results, N2I is used with four splits to the attenuation coefficient and with two splits to the electron density, labeled with *N2I 4s* and *N2I 2s* in Table 2 and 3. The uncertainties listed in Table 2 and 3 are the standard deviations over all slices from the investigated volume.

**Figure 5:**
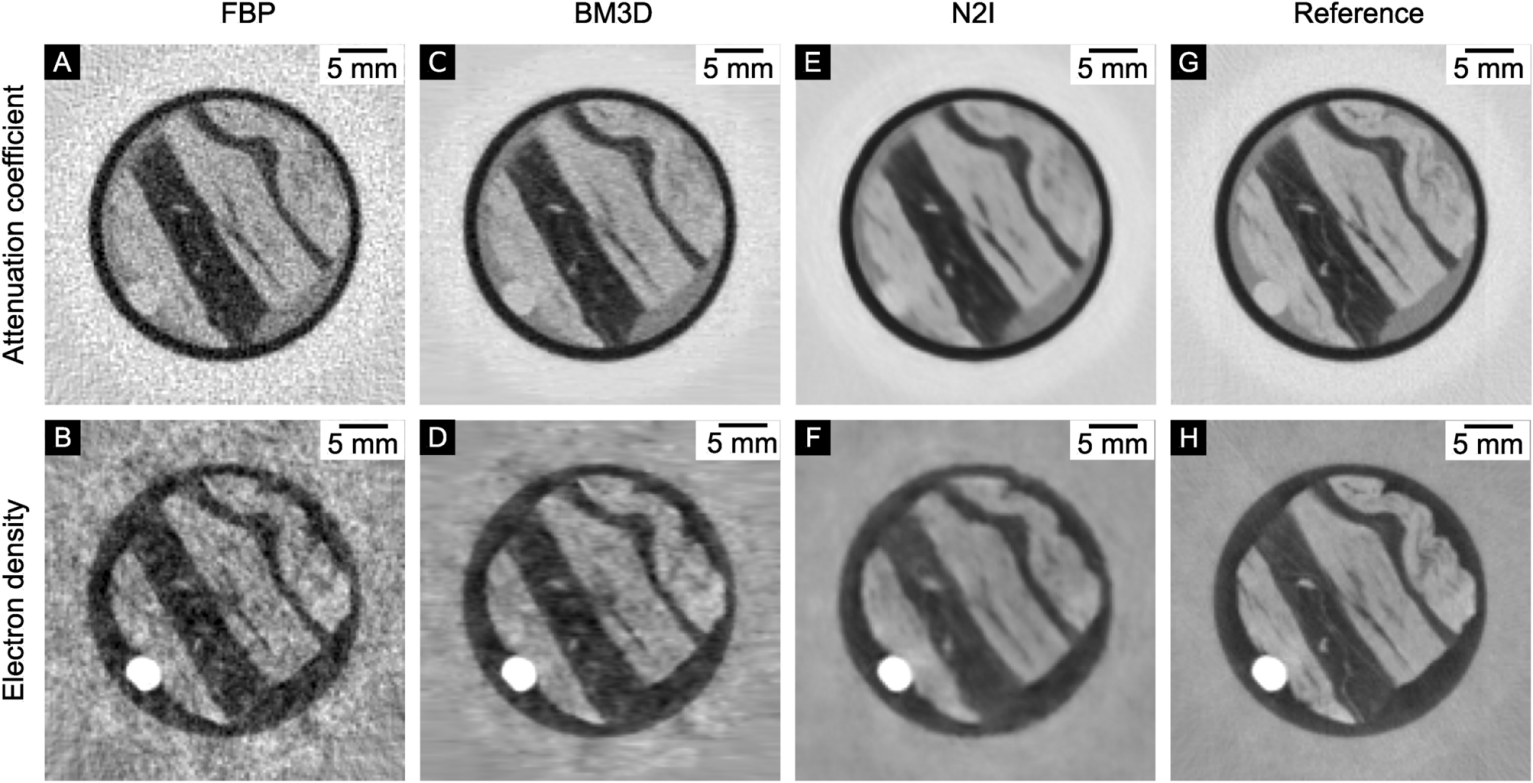
Denoising results for a dose of $20\,\hbox {mGy}$. (**A**, **B**) The non-denoised FBP. (**C**, **D**) The BM3D denoising results, (**E**, **F**) The N2I results and (**G**, **H**) the high dose reference images. It is visible, that BM3D struggles with the low-frequency noise in the electron density slice. N2I is able to drastically reduce high- and low-frequency noise. In case of the electron density slice this results in a drastic reduction of visual impairment and enables the detection of small features. However, compared to the reference slice, N2I leads to a slight blur, which is more prominent in the case of the electron density than in the case of the attenuation coefficient. The windowing for the attenuation coefficient is: [−3.5, 0.5] $\times$ 10^-3^; for the electron density: [−1.5, 1.0] $\times$ 10^-2^.

Looking at Table 2, N2I delivers the highest image quality for both the attenuation coefficient and the electron density. It is able to improve the image quality substantially compared to the normal FBP. While BM3D delivers similar PSNR values for the attenuation coefficient, N2I shows better SSIM values. The sharpness values of Table 3 indicate that N2I also delivers the best EPRr, meaning it can effectively reduce the impact of false structures introduced through the noise. However, compared to BM3D, N2I shows a lower edge accuracy (EPRa). For the electron density it even results in the lowest EPRa value. This is noticeable in a visible reduction of sharpness (see Fig. 5F, H). The sharpness of the N2I result increases with a higher number of epochs but causes overfitting of noise, and therefore the image quality decreases. This might improve for larger data sets since the network is only trained on the available 49 slices in this comparison.

BM3D shows good results but cannot compete with the IQA values of N2I. While the PSNR values are similar for the attenuation coefficient, the SSIM is still higher for N2I. In the case of the electron density, the differences are more substential, and the result shows remaining low-frequency noise, which still visually interferes with the sample structure. BM3D can maintain a decent sharpness for the attenuation coefficient and the electron density.

SIR can improve the quality beyond PPR and FBP but at the expense of image sharpness and edge accuracy, resulting in a reduction of the EPRa values compared to the normal FBP.

The use of PPR improves image quality while decreasing the sharpness only slightly for the electron density. Hence, the use of PPR is recommended when the data is reconstructed by a FBP and no other denoiser is used.

**Table 2:** Image quality values for denoiser comparison at $20\,\hbox {mGy}$.

Signal type	Method	PSNR	SSIM
Attenuation coefficient	N2I 4s	$\mathbf {27.15 \pm 0.12}$	$\mathbf {0.929 \pm 0.003}$
	BM3D	$\mathbf {27.06 \pm 0.33}$	$0.881 \pm 0.004$
	SIR	$26.29 \pm 0.24$	$0.871 \pm 0.007$
	PPR	$19.81 \pm 0.09$	$0.727 \pm 0.011$
	FBP	$18.27 \pm 0.08$	$0.681 \pm 0.011$
Electron density	N2I 2s	$\mathbf {28.81 \pm 0.56}$	$\mathbf {0.910 \pm 0.005}$
	BM3D	$25.34 \pm 0.62$	$0.843 \pm 0.008$
	SIR	$26.39 \pm 0.29$	$0.841 \pm 0.004$
	PPR	$22.63 \pm 0.51$	$0.706 \pm 0.095$
	FBP	$21.55 \pm 0.46$	$0.666 \pm 0.011$

Preferable values are marked in bold.

**Table 3:** Image sharpness values for denoiser comparison at $20\,\hbox {mGy}$.

Signal type	Method	EPRa	EPRr
Attenuation coefficient	N2I 4s	$0.746\pm 0.027$	$\mathbf {0.824 \pm 0.019}$
	BM3D	$\mathbf {0.770 \pm 0.028}$	$0.775 \pm 0.024$
	SIR	$0.683 \pm 0.020$	$0.732 \pm 0.023$
	PPR	$0.764 \pm 0.018$	$0.547 \pm 0.029$
	FBP	$0.749 \pm 0.019$	$0.503 \pm 0.034$
Electron density	N2I 2s	$0.559 \pm 0.029$	$\mathbf {0.679 \pm 0.032}$
	BM3D	$\mathbf {0.672 \pm 0.034}$	$0.470 \pm 0.046$
	SIR	$0.618 \pm 0.029$	$0.592 \pm 0.047$
	PPR	$0.659 \pm 0.026$	$0.223 \pm 0.017$
	FBP	$0.642 \pm 0.023$	$0.202 \pm 0.015$

Preferable values are marked in bold.

## Impact on medical imaging

**Figure 6:**
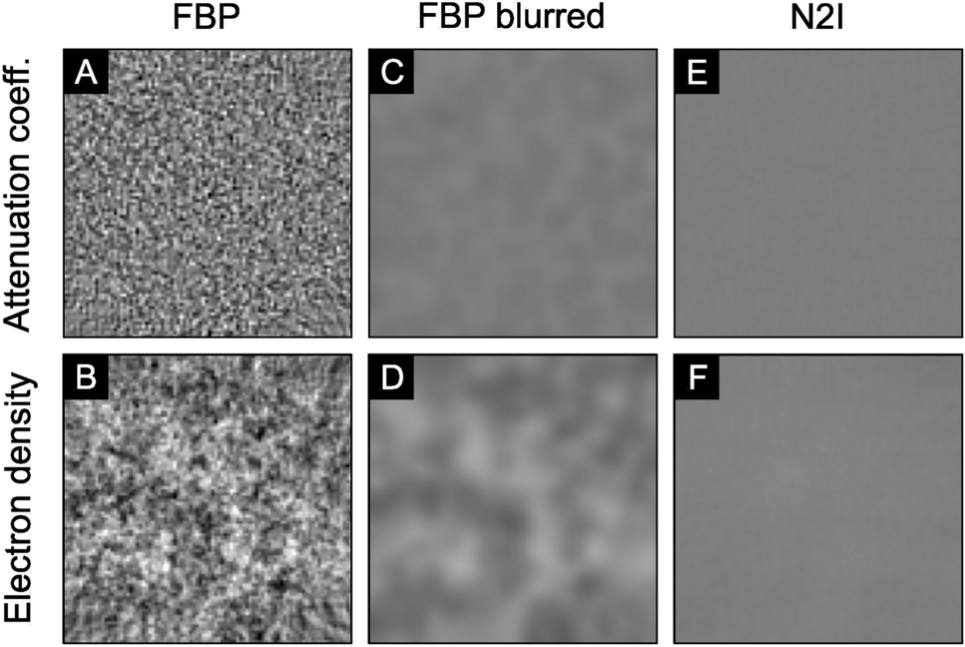
Change in noise pattern due to denoising. The noise pattern of the attenuation coefficient (**A**) and electron density (**B**) as well as the change through blurring (**C**,**D**) and N2I (**E**,**F**). While the noise in the attenuation coefficient can be efficiently reduced through blurring (**C**), a visually disruptive noise pattern remains for the electron density (**D**). N2I can effectively reduce the noise for both signal types (**E**,**F**). The values are normalized between 0 and 1. The windowing is: [0.2, 0.8] for both signals.

Raupach and Flohr^12^ developed a mathematical framework to analyze the maximal theoretical performance of gbPC-CT by evaluating the ratio between phase and absorption contrast. They mention that gbPC-CT has a fundamentally different noise power spectrum (NPS) than the conventional abCT, which results in a resolution dependency of its performance^10–12^. For low-resolution imaging at full-body CT, the higher contrast of the phase can therefore be overcompensated by the low coherence lengths of gbPC-CT setups with low brilliant sources, resulting in a worse dose efficiency than abCT. The relative dose efficiency improves with increasing resolution. An established method to further reduce the total noise power is the usage of smoothing filters, which decrease the spatial resolution. Such methods are less effective for gbPC-CT, which has a higher noise power in the low-frequencies compared to abCT, as Raupach and Flohr^12^ showed on simulated data. This fact is replicated with experimental data, where a measurement without a sample is performed and reconstructed. The result is seen in Fig. 6.

**Figure 7:**
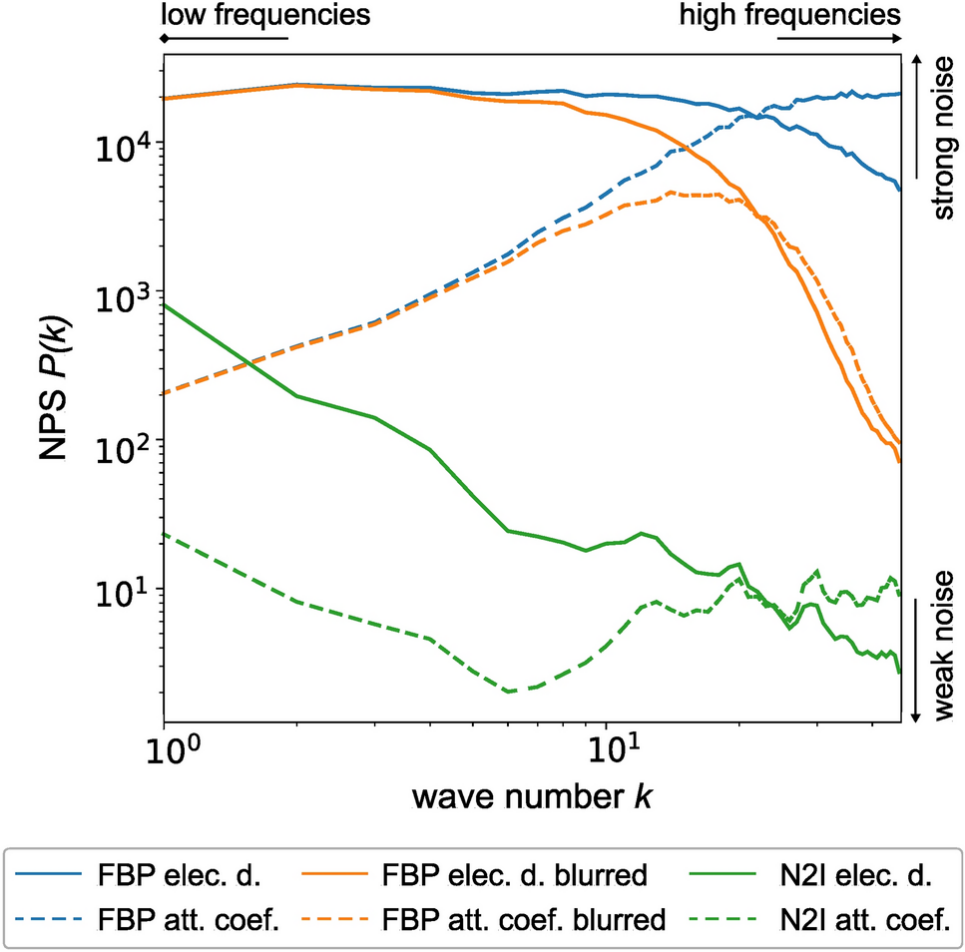
Change in NPS due to denoising. The NPS *P*(*k*) over the wave number *k* for the FBP, the application of blur, and N2I. Blurring reduces the high frequencies effectively but has no impact on the low frequencies, which are dominant in the electron density. N2I can reduce the noise in the low and high frequencies drastically.

The left images show the FBP. Next to it is the FBP with a subsequent Gaussian blur for noise reduction. While the noise pattern in the attenuation coefficient nearly disappears, there are still noticeable remaining noise structures in the electron density. However, this changes with the use of N2I. Since N2I is trained on the corresponding data, it also manages to effectively reduce the low-frequency noise in the electron density, resulting in a smooth image. The change of the NPS due to the application of N2I can be seen in Fig. 7. The differences between the attenuation coefficient and the electron density are clearly visible. The Gaussian blur reduces the high frequencies in both cases effectively, but it is visible that the main part of the electron density NPS is in the lower frequencies, which are not impacted by the blur, resulting in less quality improvement than for the attenuation coefficient. The application of N2I drastically reduces the high- and low-frequency noise in both cases. Nevertheless, the low frequencies of the electron density are still slightly higher and there is a small remaining peak in the high-frequency of the attenuation coefficient. N2I can therefore drastically reduce the impact of the effects introduced through the different NPS, resulting in a strong quality improvement of the reconstruction, which is valid for experimental data as seen in Fig. 5.

## Discussion

We showed that for the application of N2I on the electron density, the choice of two splits is preferred since small structures in the muscle tissue are easier to detect. However, for its application on the attenuation coefficient, all IQA values show better numbers when used with four splits. The quality of N2I could be further improved by investigating the influence of other hyper parameters. One of them is the learning strategy. It describes how many of the splits are used as input during training. The X:1 strategy uses all but one split as input. N2I applied on abCT data achieves the highest image quality when trained with the X:1 strategy and four splits^16^. But since the electron density images show different behavior for the X:1 strategy, it seems reasonable to test the 1:X strategy on electron density data.

N2I showed strong image quality improvements while trained only on one single noisy tomogram. Therefore, it does not require high-dose reference measurements, which are difficult to obtain in the field of medical imaging. Following the argumentation of Raupach and Flohr^12^, a decrease in the low-frequency noise and thus an increase in the CNR of gbPC-CT leads to a lower break-even point of gbPC-CT and abCT performance, which could lead to a higher dose efficiency of gbPC-CT compared to abCT. Put differently, using N2I allows the use of higher resolution while maintaining a CNR that is necessary for diagnosis, where gbPC-CT is able to outperform the conventional abCT in terms of dose. This is supported by a recent study by Rawlik et al.^11^. They showed, based on experimental data, that the break-even point of gbPC-CT and abCT for a fixed CNR of five lays at a resolution of 214 mm with a dose of 65 mGy. The use of N2I allows either to lower the dose, maintaining the CNR and resolution, or increasing the resolution, maintaining the CNR and dose. In both cases, N2I shifts the break-even point in favor of gbPC-CT. Even though N2I can handle low-frequency noise, a deeper understanding of the interaction between N2I and small structures with varying contrast is required for medical applications. To further investigate the properties of N2I, simulations containing objects of different sizes, compositions, and shapes could be run. Various noise models, with high- and low-frequency noise, could be applied to these simulations while observing the behavior of N2I. This should give a better understanding of its impact on the resolution of small structures. Experimental measurements with phantoms of known structure sizes and material composition could afterward validate these results. While BM3D delivers similar performance when applied to the attenuation coefficient, it struggles with the low-frequency noise in the electron density slices. This visualizes the problem of many non-neural-network-based denoisers. They are mainly optimized for the pixel-wise noise apparent in projections and, therefore, struggle with the different noise characteristics in the electron density slices.

N2I can maintain the sharpness for the attenuation coefficient. However, when applied to the electron density, it lacks sharpness. This might be mitigated by training the model on larger data sets. The results also heavily depend on the choice of the neural network architecture. We utilized the MS-D network with small parameter numbers as suggested by^16^ since it decreases the risk of over-fitting, and only small samples with a limited number of pixels and slices were measured. However, there might be more sophisticated networks that can achieve better results for gbPC-CT measurements since CNNs have inductive biases. One example is the size of the convolutional kernels, which are set by the chosen structure of the network and are not trained on the data itself. While this helps the CNN to achieve high performance with minimal data, it limits the model when trained on a large quantity of data. In such cases, transformers with minimal inductive biases can outperform CNNs^46^ and should be considered when building a persistent model. In addition, it is known that the performance of N2I is limited by the variance of the noise. Other supervised deep-learning networks trained on noisy and noise-free image pairs might outperform N2I if enough training data is available^47^.

In our paper, we trained the N2I model on each measurement and applied it afterward. However, this results in high computational efforts and is unsuitable for medical facility applications. Therefore, the next step should be to build a database that can be used to train a more general model. Based on this model, N2I would be applicable to new data without the lengthy learning process and could deliver superior results with lower computational expense than SIR. Two separate models should be created, one for the attenuation coefficient and one for the electron density, as the noise power spectrum of the two signals differs considerably.

## Conclusion

N2I allows the application of deep learning denoising in the field of gbPC-CT, where not enough data is yet available to sufficiently train supervised denoising methods. It is capable of efficiently denoising high- and low-frequency noise. Therefore, it substantially increases the image quality. This allows to push towards higher resolutions and lowers the break-even point of gbPC-CT and conventional abCT, which might ultimately lead to a higher dose efficiency of gbPC-CT. Thus, the application of self-supervised denoising methods like N2I brings gbPC-CT one step closer to medical application.

## Data Availability

The datasets generated during and/or analyzed during the current study are available from the corresponding author on reasonable request.

## References

[1] Bos, D. et al. Radiation exposure in computed tomography. *Deutsches Ärzteblatt Int.*[SPACE]10.3238/arztebl.m2022.0395 (2023). 10.3238/arztebl.m2022.0395 PMC10198168 36633449

[2] Donath, T. et al. Toward clinical X-ray phase-contrast CT. *Invest. Radiol.* **45**, 445–452. 10.1097/RLI.0b013e3181e21866 (2010). 20498610 10.1097/RLI.0b013e3181e21866

[3] Fitzgerald, R. Phase-sensitive X-ray imaging. *Phys. Today* **53**, 23–26. 10.1063/1.1292471 (2000).

[4] Momose, A. Phase-sensitive imaging and phase tomography using X-ray interferometers. *Opt. Exp.* **11**, 2303. 10.1364/OE.11.002303 (2003). 10.1364/oe.11.002303 19471338

[5] Pfeiffer, F., Weitkamp, T., Bunk, O. & David, C. Phase retrieval and differential phase-contrast imaging with low-brilliance X-ray sources. *Nat. Phys.* **2**, 258–261. 10.1038/nphys265 (2006).

[6] Stampanoni, M. et al. The first analysis and clinical evaluation of native breast tissue using differential phase-contrast mammography. *Invest. Radiol.* **46**, 801–806. 10.1097/RLI.0b013e31822a585f (2011). 21788904 10.1097/RLI.0b013e31822a585f

[7] Wang, Z. et al. Non-invasive classification of microcalcifications with phase-contrast X-ray mammography. *Nat. Commun.* **5**, 3797. 10.1038/ncomms4797 (2014). 24827387 10.1038/ncomms4797

[8] Scherer, K. et al. Correspondence: Quantitative evaluation of X-ray dark-field images for microcalcification analysis in mammography. *Nat. Commun.* **7**, 10863. 10.1038/ncomms10863 (2016). 27102865 10.1038/ncomms10863 PMC4844690

[9] Scherer, K. et al. Improved diagnostics by assessing the micromorphology of breast calcifications via X-ray dark-field radiography. *Sci. Rep.* **6**, 1–11. 10.1038/srep36991 (2016). 27841341 10.1038/srep36991 PMC5107908

[10] Chen, G.-H., Zambelli, J., Li, K., Bevins, N. & Qi, Z. Scaling law for noise variance and spatial resolution in differential phase contrast computed tomography. *Med. Phys.* **38**, 584–588. 10.1118/1.3533718 (2011). 21452695 10.1118/1.3533718 PMC3030613

[11] Rawlik, M. et al. Increased dose efficiency of breast CT with grating interferometry. *Optica* **10**, 938. 10.1364/OPTICA.487795 (2023) arXiv:2301.00455.

[12] Raupach, R. & Flohr, T. Performance evaluation of x-ray differential phase contrast computed tomography (PCT) with respect to medical imaging. *Med. Phys.* **39**, 4761–4774. 10.1118/1.4736529 (2012). 22894401 10.1118/1.4736529

[13] Chen, H. et al. Low-dose CT with a residual encoder-decoder convolutional neural network. *IEEE Trans. Med. Imaging* **36**, 2524–2535. 10.1109/TMI.2017.2715284 (2017) arXiv:1702.00288. 28622671 10.1109/TMI.2017.2715284 PMC5727581

[14] Han, Y. & Ye, J. C. Framing U-Net via deep convolutional framelets: Application to sparse-view CT. *IEEE Trans. Med. Imaging* **37**, 1418–1429. 10.1109/TMI.2018.2823768 (2018) arXiv:1708.08333. 29870370 10.1109/TMI.2018.2823768

[15] Shan, H. et al. 3-D convolutional encoder-decoder network for low-dose CT via transfer learning from a 2-D trained network. *IEEE Trans. Med. Imaging* **37**, 1522–1534. 10.1109/TMI.2018.2832217 (2018) arXiv:1802.05656. 29870379 10.1109/TMI.2018.2832217 PMC6022756

[16] Hendriksen, A. A., Pelt, D. M. & Batenburg, K. J. Noise2Inverse: Self-supervised deep convolutional denoising for tomography. *IEEE Trans. Comput. Imaging* **6**, 1320–1335. 10.1109/TCI.2020.3019647 (2020) arXiv:2001.11801.

[17] Flenner, S. et al. Machine learning denoising of high-resolution X-ray nanotomography data. *J. Synchrotron Radiat.* **29**, 230–238. 10.1107/S1600577521011139 (2022). 34985440 10.1107/S1600577521011139 PMC8733986

[18] Berger, N. et al. Dedicated breast computed tomography with a photon-counting detector: Initial results of clinical in vivo imaging. *Invest. Radiol.* **54**, 409–418. 10.1097/RLI.0000000000000552 (2019). 30829942 10.1097/RLI.0000000000000552

[19] Oostveen, L. J. et al. Physical evaluation of an ultra-high-resolution CT scanner. *Eur. Radiol.* **30**, 2552–2560. 10.1007/s00330-019-06635-5 (2020). 32040726 10.1007/s00330-019-06635-5 PMC7160079

[20] Willner, M. et al. Quantitative breast tissue characterization using grating-based X-ray phase-contrast imaging. *Phys. Med. Biol.* **59**, 1557–1571. 10.1088/0031-9155/59/7/1557 (2014). 24614413 10.1088/0031-9155/59/7/1557

[21] Birnbacher, L. et al. Experimental realisation of high-sensitivity laboratory X-ray grating-based phase-contrast computed tomography. *Sci. Rep.* **6**, 24022. 10.1038/srep24022 (2016). 27040492 10.1038/srep24022 PMC4819174

[22] Pfeiffer, F. et al. Hard-X-ray dark-field imaging using a grating interferometer. *Nat. Mater.* **7**, 134–137. 10.1038/nmat2096 (2008). 18204454 10.1038/nmat2096

[23] Mechlem, K., Sellerer, T., Viermetz, M., Herzen, J. & Pfeiffer, F. A theoretical framework for comparing noise characteristics of spectral, differential phase-contrast and spectral differential phase-contrast X-ray imaging. *Phys. Med. Biol.* **65**, 065010. 10.1088/1361-6560/ab7106 (2020) arXiv:1910.00899. 31995518 10.1088/1361-6560/ab7106

[24] Raupach, R. & Flohr, T. G. Analytical evaluation of the signal and noise propagation in X-ray differential phase-contrast computed tomography. *Phys. Med. Biol.* **56**, 2219–2244. 10.1088/0031-9155/56/7/020 (2011). 21403187 10.1088/0031-9155/56/7/020

[25] Köhler, T., Jürgen Engel, K. & Roessl, E. Noise properties of grating-based X-ray phase contrast computed tomography. *Med. Phys.* **38**, S106–S116. 10.1118/1.3532396 (2011). 21978111 10.1118/1.3532396

[26] Mitos GmbH. *X-aid*.

[27] Boone, J. M. Normalized glandular dose (DgN) coefficients for arbitrary X-ray spectra in mammography: Computer-fit values of Monte Carlo derived data. *Med. Phys.* **29**, 869–875. 10.1118/1.1472499 (2002). 12033583 10.1118/1.1472499

[28] Eggl, E. et al. Dose-compatible grating-based phase-contrast mammography on mastectomy specimens using a compact synchrotron source. *Sci. Rep.* **8**, 15700. 10.1038/s41598-018-33628-z (2018). 30356116 10.1038/s41598-018-33628-z PMC6200806

[29] Bundesamt für Strahlenschutz. BfS—Limit values in radiation protection. Accessed 11 Dec 2023 (2022).

[30] Haeusele, J. et al. Advanced phase-retrieval for stepping-free X-ray dark-field computed tomography. *IEEE Trans. Med. Imaging* 1–1. 10.1109/TMI.2023.3271413 (2023). 10.1109/TMI.2023.3271413 37115841

[31] Dabov, K., Foi, A., Katkovnik, V. & Egiazarian, K. Image denoising by sparse 3-D transform-domain collaborative filtering. *IEEE Trans. Image Process.* **16**, 2080–2095. 10.1109/TIP.2007.901238 (2007). 17688213 10.1109/tip.2007.901238

[32] Mäkinen, Y., Azzari, L. & Foi, A. Exact transform-domain noise variance for collaborative filtering of stationary correlated noise. In *IEEE International Conference on Image Processing*. 185–189 (2019). 10.1109/TIP.2020.3014721 32784137

[33] Fumene Feruglio, P., Vinegoni, C., Gros, J., Sbarbati, A. & Weissleder, R. Block matching 3D random noise filtering for absorption optical projection tomography. *Phys. Med. Biol.* **55**, 5401–5415. 10.1088/0031-9155/55/18/009 (2010) arXiv:NIHMS150003. 20736500 10.1088/0031-9155/55/18/009 PMC2934766

[34] Sheng, K., Gou, S., Wu, J. & Qi, S. X. Denoised and texture enhanced MVCT to improve soft tissue conspicuity. *Med. Phys.* **41**, 101916. 10.1118/1.4894714 (2014). 25281968 10.1118/1.4894714

[35] Mäkinen, Y., Azzari, L. & Foi, A. Collaborative filtering of correlated noise: Exact transform-domain variance for improved shrinkage and patch matching. *IEEE Trans. Image Process.* **29**, 8339–8354. 10.1109/TIP.2020.3014721 (2020). 10.1109/TIP.2020.3014721 32784137

[36] Batson, J. & Royer, L. Noise2Self: Blind denoising by self-supervision. In *36th International Conference on Machine Learning, ICML 2019*. 826–835. 10.48550/arXiv:.1901.11365. arXiv:1901.11365 (2019).

[37] Pelt, D. M. & Sethian, J. A. A mixed-scale dense convolutional neural network for image analysis. *Proc. Natl. Acad. Sci.* **115**, 254–259. 10.1073/pnas.1715832114 (2018). 29279403 10.1073/pnas.1715832114 PMC5777062

[38] Nyquist, H. Certain topics in telegraph transmission theory. *Trans. Am. Inst. Electr. Eng.* **47**, 617–644. 10.1109/T-AIEE.1928.5055024 (1928).

[39] Shannon, C. Communication in the presence of noise. *Proc. IRE* **37**, 10–21. 10.1109/JRPROC.1949.232969 (1949).

[40] Crowther, R. A., Derosier, D. J. & Klug, A. The reconstruction of a three-dimensional structure from projections and its application to electron microscopy. *Proc. R. Soc. Lond. A Math. Phys. Sci.* **317**, 319–340. 10.1098/rspa.1970.0119 (1970).

[41] Sara, U., Akter, M. & Uddin, M. S. Image quality assessment through FSIM, SSIM, MSE and PSNR-A comparative study. *J. Comput. Commun.* **07**, 8–18. 10.4236/jcc.2019.73002 (2019).

[42] Sonawane, S. & Prof. Deshpande, A. M. Image quality assessment techniques: An overview. *Int. J. Eng. Res. Technol. (IJERT)* **03** (2014).

[43] Wang, Z., Bovik, A., Sheikh, H. & Simoncelli, E. Image quality assessment: From error visibility to structural similarity. *IEEE Trans. Image Process.* **13**, 600–612. 10.1109/TIP.2003.819861 (2004). 15376593 10.1109/tip.2003.819861

[44] Chen, L. et al. Edge preservation ratio for image sharpness assessment. In *2016 12th World Congress on Intelligent Control and Automation (WCICA)*. Vol. 2016. 1377–1381. 10.1109/WCICA.2016.7578241 (IEEE, 2016).

[45] Canny, J. A Computational approach to edge detection. *IEEE Trans. Pattern Anal. Mach. Intell.* **PAMI-8**, 679–698. 10.1109/TPAMI.1986.4767851 (1986). 21869365

[46] D’Ascoli, S. et al. ConViT: Improving vision transformers with soft convolutional inductive biases. *J. Stat. Mech. Theory Exp.* **2022**, 114005. 10.1088/1742-5468/ac9830 (2022) arXiv:2103.10697.

[47] Lehtinen, J. et al. Noise2Noise: Learning image restoration without clean data. In *35th International Conference on Machine Learning ICML 2018*. Vol. 7. 4620–4631. arXiv:1803.04189 (2018) .

